# Dot map cartograms for detection of infectious disease outbreaks: an application to Q fever, the Netherlands and pertussis, Germany

**DOI:** 10.2807/1560-7917.ES.2017.22.26.30562

**Published:** 2017-06-29

**Authors:** Loes Soetens, Susan Hahné, Jacco Wallinga

**Affiliations:** 1 Centre for Infectious Disease Control, National Institute for Public Health and the Environment, Bilthoven, The Netherlands; 2 Department of Medical Statistics, Leiden University Medical Centre, Leiden, The Netherlands

**Keywords:** Outbreaks, Spatial methods, Maps, Geographical Information Systems, Q fever, pertussis

## Abstract

Geographical mapping of infectious diseases is an important tool for detecting and characterising outbreaks. Two common mapping methods, dot maps and incidence maps, have important shortcomings. The former does not represent population density and can compromise case privacy, and the latter relies on pre-defined administrative boundaries. We propose a method that overcomes these limitations: dot map cartograms. These create a point pattern of cases while reshaping spatial units, such that spatial area becomes proportional to population size. We compared these dot map cartograms with standard dot maps and incidence maps on four criteria, using two example datasets. Dot map cartograms were able to illustrate both incidence and absolute numbers of cases (criterion 1): they revealed potential source locations (Q fever, the Netherlands) and clusters with high incidence (pertussis, Germany). Unlike incidence maps, they were insensitive to choices regarding spatial scale (criterion 2). Dot map cartograms ensured the privacy of cases (criterion 3) by spatial distortion; however, this occurred at the expense of recognition of locations (criterion 4). We demonstrate that dot map cartograms are a valuable method for detection and visualisation of infectious disease outbreaks, which facilitates informed and appropriate actions by public health professionals, to investigate and control outbreaks.

## Introduction

Here we propose a method of mapping infectious disease data, the dot map cartogram, which displays the geographical locations of reported cases from routine surveillance or outbreak investigations, such that public health experts can visualise both absolute numbers and spatial trends in incidence of infection. The method is developed to address two major causes of misinterpretation in commonly used maps: masking patterns of disease by not taking into account the population density distribution, and masking patterns by categorisation of information (across space and incidence of disease). With the dot map cartogram, we address the problem, raised by a recent systematic review [1], that spatial methods are underutilised and used in only ca 0.4% of all published outbreak investigations.

The two most frequently used map types for spatial outbreak data are the dot map and choropleth incidence map [1]. In a dot map, every case is represented by a point on the map, showing absolute numbers of cases. Illustrating absolute numbers of cases reveals the size of the outbreak and identifies areas without cases. Dot maps do not account for the underlying geographical distribution of the population. As populations are usually heterogeneously distributed, important variations in incidence of infection can be masked. Another drawback of dot maps is that they may reveal too much information about the location of specific cases, by which the privacy of a case might be violated.

In a choropleth map, a quantitative attribute is displayed per spatial unit. For example, ordinal classes of incidence may be displayed by municipality, with the areas shaded according to their incidence value and a range of shading classes [2]. Choropleth maps are mostly used to display surveillance data. They are less suitable to display outbreak data when the interest is in exact locations of cases. Although this type of map gives a quick overview of where there are more cases than expected based on the background population, it has a major limitation: The appearance of this map is heavily influenced by arbitrary choices with regard to the classification system for the ordinal classes and the spatial unit used (the latter is also referred to as the Modifiable Areal Unit Problem, MAUP [3]). Because the data are categorised both across the quantitative attribute and across the geographical units, this can lead to a loss of information by masking important internal patterns and variations. Therefore, this type of map can be misleading. This is especially problematic when mapping rare events, which is often the case with infectious diseases.

Here we combine the advantages of both maps into one map type which we call dot map cartogram. This map type uses a diffusion-based algorithm [4], which creates contiguous or value-by-area cartograms. In these value-by-area cartograms, regions are enlarged or reduced relative to the number of individuals they contain. We apply this principle to traditional dot maps: we deform the map contours based on population size and simultaneously deform the dot pattern. In this way, a dense point pattern in a big city will become more dispersed, whereas a dense point pattern in a rural area will become even more dense. A similar technique called density-equalising map projection (DEMP) has been pioneered before to describe the spatial distribution of cryptosporidiosis among AIDS patients in San Francisco [5]. This earlier study used a different density-equalising algorithm, and application was limited to local outbreak data. This method has the advantages that the density of the dots reflects the incidence, the number of dots represent the absolute number of cases, and arbitrary choices for scale of spatial unit and classification system are avoided.

In this study, we assessed whether the advantages of the dot map cartogram outweigh the potential disadvantages. One potential drawback is that the dot map cartogram may reveal too much information with regard to the privacy of the cases. Another potential drawback is that the spatial distortion makes it hard to recognise or localise the map topology. We created dot map cartograms using real life data and compared them to the traditional choropleth and dot maps regarding four criteria: (i) ability to show both absolute numbers and incidence of the disease, (ii) sensitivity to choices regarding spatial scale and classification system, (iii) ability to ensure the privacy of individual cases, and (iv) ability to recognise locations. The comparison was applied to the mapping of a point-source outbreak and to the mapping of the occurrence of a human-to-human transmissible disease.

## Methods

### Data

As an example of outbreak mapping to locate a source, we used data from the Q fever outbreak in the Netherlands in 2009. Q fever case reports in the Netherlands communicable disease notification system (Osiris) include the 4-digit postal code of residence. Cases with date of onset of illness in 2009 (n = 1,740) were extracted for this analysis. Population data and shapefiles (a data format which stores geometrical locations and metadata such as population size per geometrical location) on different spatial levels (4-digit postal code and municipality level) were accessed via Statistics Netherlands [5].

As an example of a human-to-human transmissible disease, we used pertussis notifications in Germany in 2015. Data on pertussis cases was made available by the Robert Koch Institute through the SurvStat@RKI 2.0 web portal [6], in which the district of residence is registered for every case. We extracted data on laboratory-confirmed pertussis cases with a date of diagnosis in 2015 (n = 9,017). The most recent population data and shapefiles for district and federal state level boundaries (2013) were accessed through the open data portal of the Federal Agency for Cartography and Geodesy Germany [7]. We assumed that the population size per district or federal state in 2015 was sufficiently similar to that in 2013.

### Geographical representation

#### Dot map

In a dot map, every case is represented by a dot on their location of residence. As this would reveal the exact locations of the cases and harm their privacy, we chose to use a proxy location by assigning a random point location in the spatial unit of residence (4-digit postal code for the Netherlands and district for Germany) to every case. The 4-digit postal code areas in the Netherlands have a mean population of 4,119 persons (mean surface: 8.6 km^2^), and the districts in Germany have a mean population of 200,914 persons (mean surface: 888.7 km^2^). For the map of the Netherlands we have used the national RD (‘rijksdriehoeksstelsel’)-based projection and for Germany we used the Universal Transverse Mercator (UTM) 32 projection. Both are conformal map projections in which local angles are preserved and shapes are represented accurately and without distortion for small areas.

#### Choropleth incidence map

In a choropleth incidence map, disease incidence is displayed per spatial unit, using ordinal classes. We chose two spatial unit levels for each country: one at the same level as the patient data (spatial unit of residence as described above) and the other one level higher (municipality in the Netherlands or federal state in Germany). In addition, we categorised incidence into five ordinal classes, using two separate classification systems: the Jenks’ natural breaks algorithm [8] which seeks to reduce the variance within classes and maximise the variance between classes, and the quantile method in which equal numbers of spatial units are placed into each class [8]. The colour schemes for the incidence classes were based on previously established map colour palettes [9].

#### Dot map cartogram

##### Basic principle

We created cartograms by reshaping the spatial units such that their area was proportional to their population by applying the Gastner-Newman diffusion-based algorithm [4] without changing the underlying map topology [10]. The principle is that once the areas have been scaled to be proportional to their population, then population density is by definition the same for each area of visually the same size on the cartogram. To create dot map cartograms, the point patterns of cases (as described in the dot map section) were simultaneously transformed in the reshape process of the spatial units so that the number of cases per unit area reflected the incidence. In addition, to provide points of reference to interpret the dot map cartograms, capital municipality, provincial or state boundaries were simultaneously transformed, and an inset was added with the undistorted map. We used ScapeToad 1.2 software [11] to reshape the spatial units and the point patterns. From this programme, the transformed layers were exported as shapefiles. The exported files were imported into R statistical software to create the maps. The main R package used to create the maps was *ggplot2*. The R code for the construction of the dot map cartogram is provided elsewhere (https://github.com/lsoetens/DotMapCartogram).

##### Cartogram quality

As we used spatial units with a population size at a certain spatial level to deform the original map, the MAUP problem was inherited. However, the consequences of this problem can be reduced by relying on an objective measure to assess which spatial level we could best use for our transformations. We assessed the cartogram quality by the objective measures, i.e. the average and maximum normalised cartographic error [10]. The cartographic error is the most commonly used measure for distortion in the value-by-area realisation. We assumed an input map *M* that is partitioned into *n* regions with polygonal boundaries. For each region *v*, *a(v)* denotes the area of *v* in *M* and the weight *w(v)* is the desired area for the region based on the background population. The diffusion-based algorithm constructed the cartogram *M^0^*, that is a deformation of *M*, in which *o(v)* is the observed area of region *v*. The average normalised cartographic error *e* was calculated as:

$$e=\frac{1}{n}\sum_{v\in V}\frac{\left|o(v)-w(v)\right|}{\max\left\{o(v),w(v)\right\}}$$

with a range of [0,1] and the maximum normalised cartographic error *x* was calculated as:

$$x=\max\left(\frac{\left|o(v)-w(v)\right|}{\max\left\{o(v),w(v)\right\}}\right)$$

with a range of [0,1]

No guidelines exist as to what constitutes acceptable cartogram quality; to be consistent with the values measured in a previous publication [10], we aimed for *e* < 0.1 and *x* < 0.4 in our cartograms. The performance of the algorithm, and subsequently the cartogram quality, depends on the distribution of the population density in the input file and the grid size on which the computation of the population density is based. Large discrepancies in the population density between regions impair the algorithm performance, while a finer grid will produce a higher quality cartogram, but may introduce distortion. We started with an input map at the same spatial unit of residence as the case data (i.e. 4-digit postal code for the Netherlands and district level for Germany) and assessed the average and maximum error of the cartograms. For the Netherlands, cartogram error was not satisfactory and we changed to the municipality level and accepted a maximum normalised cartogram error of *x* > 0.4; this was due to the very small population size of the municipalities on the West Frisian Barrier Islands. These areas should be smaller than presented in the created maps, but would no longer be visible. We used a 512 × 512 grid, which gave sufficient results; a higher-resolution grid of 1,024 × 1,024 is possible, but requires substantially greater computation times.

### User requirements

To create the dot map cartograms, no specific geographical information system skills are needed. The only requirement is some basic knowledge of the R statistical software to be able to run the provided code (https://github.com/lsoetens/DotMapCartogram). Required software includes R studio and ScapeToad [11]. Both programmes are open source programmes, which can be downloaded for free. A detailed manual for creating the dot map cartograms is provided along with the code (https://github.com/lsoetens/DotMapCartogram).

## Results

### Geographical representation of point-source outbreaks: Q fever in the Netherlands

**Figure 1:**
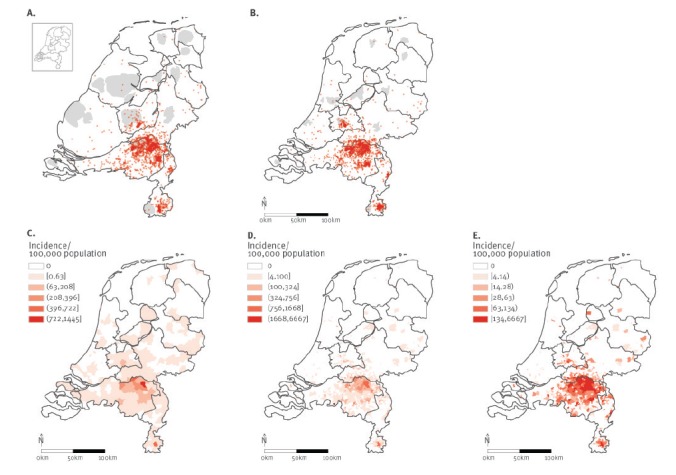
Spatial mapping of the Q fever outbreak in the Netherlands, 2009

The maps of Q fever cases are compared in Figure 1. The dot map cartogram (Figure 1A) is based on the population size per municipality in the Netherlands in 2009 (*e* = 0.08, sd = 0.09; *x* = 0.78). It is compared with the dot map (Figure 1B) and the choropleth incidence maps (Figures 1C-E).

A. Dot map cartogram. One red dot represents one case, shaded grey areas indicate the national and provincial capital municipalities. The geographical administrative units displayed as underlying layer are distorted as a result of building the cartograms.

B. Dot map. One red dot represents one case, shaded grey areas indicate the national and provincial capital municipalities.

C. Choropleth incidence map by municipality and Jenks’ natural breaks classification system.

D. Choropleth incidence map by 4-digit postal code and Jenks’ natural breaks classification system.

E. Choropleth incidence map by 4-digit postal code and the quantile classification system.

#### 

##### Ability to show both absolute numbers and incidence

The dot map cartogram is able to show both absolute numbers and incidence. Incidence is readily inferred from the choropleth incidence maps, but the exact size of the outbreak in absolute numbers is not apparent. The dot map showed a clear clustering pattern with several hotspots, but whether this is related to the population density (incidence) cannot be inferred from the dot map.

##### Sensitivity to choices regarding spatial scale and classification system

The dot map cartogram and dot map do not suffer from arbitrary classification issues. The choropleth incidence maps are sensitive to this; different spatial scale and classification systems result in highly variable maps. When only one of those maps is used, the geography of the outbreak could be misinterpreted. One would conclude that the outbreak is more widespread (Figure 1C) or more intense (Figure 1E) than is illustrated in Figure 1D.

##### Ability to ensure the privacy of cases

The dot map compromises case privacy by revealing exact locations of cases, while the choropleth incidence map protects privacy by aggregating case information. The dot map cartogram meets this criterion partially by deforming the underlying region.

##### Ability to recognise locations

The dot map cartogram is harder to read, and specific locations are difficult to recognise. This can be improved by providing locations of major towns or district boundaries. The choropleth incidence map and dot map outperform the dot map cartogram on this criterion.

### Geographical representation of a human-to-human transmissible disease: pertussis in Germany

**Figure 2:**
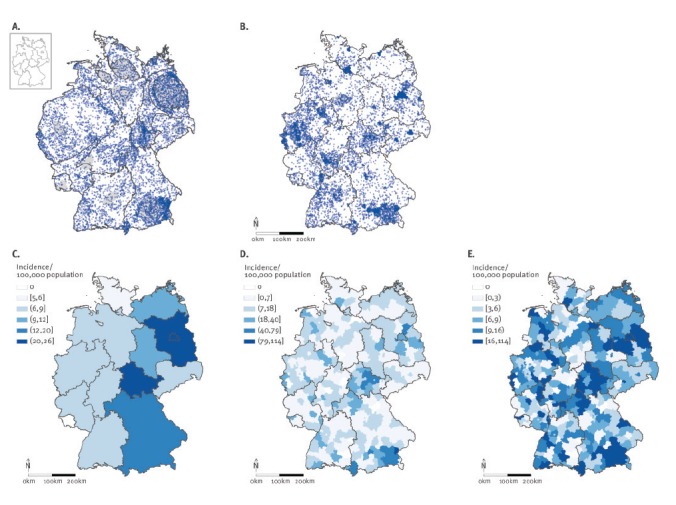
Spatial mapping of endemic pertussis in Germany, 2015

The maps for pertussis cases are compared in Figure 2. Figure 2A depicts the dot map cartogram based on the population size by district in Germany in 2013 (*e* = 0.07, sd = 0.06; *x* = 0.29); this map is contrasted with the corresponding dot map (Figure 2B) and choropleth incidence maps (Figures 2C-E).

A. Dot map cartogram. One blue dot represents one case, shaded grey areas indicate the national and federal capital municipalities. The geographical administrative units displayed as underlying layer are distorted as a result of building the cartograms.

B. Dot map. One blue dot represents one case, shaded grey areas indicate the national and federal capital municipalities.

C. Choropleth incidence map by federal state and Jenks’ natural breaks classification system.

D. Choropleth incidence map by district and Jenks’ natural breaks classification system.

E. Choropleth incidence map by district and the quantile classification system.

#### 

##### Ability to show both absolute numbers and incidence

A much larger number of cases are illustrated in this example than for the Q fever data. This is immediately apparent from the dot map and the dot map cartogram, but less so from the choropleth incidence map. The clustering patterns on the dot map in several large cities and for example in the Ruhr district (in the west of Germany) disappear into an almost random pattern in the dot map cartogram, indicating that the clustering patterns on the dot map can be explained by the underlying population density in those areas. In contrast, in the south-east of Germany near Munich, a clustering pattern is present in both the dot map and the dot map cartogram, indicating that this cluster is not attributable to population density but reflects a higher incidence of pertussis notifications. Therefore, in this situation, both incidence and absolute number of cases are important in interpreting the map: they reveal the size of the problem and provide clustering of cases not attributable to the population density.

##### Sensitivity to choices regarding spatial scale and classification system

The choropleth incidence maps showed that the choice of classification system or spatial level results in highly variable maps. In this comparison, Figure 2D, based on the Jenks’ natural breaks classification system and the district level, is comparable to the dot map cartogram, showing the same areas with high incidence; however, there is no way to determine a priori which classification system and spatial level would result in the ‘right’ map.

##### Ability to secure the privacy of cases

All maps meet this criterion. In this dataset, the dot map does not compromise privacy because of the frequency of occurrence of the disease. As before, the dot map cartogram protects case privacy due to the deformation of the underlying regions, and the choropleth incidence map because information is aggregated.

##### Ability to recognise locations

The choropleth incidence map and dot map outperform the dot map cartogram on this criterion. Adding reference points in the dot map cartogram, such as the federal state and federal state municipality boundaries, can help in recognising locations.

## Discussion

We have proposed the dot map cartogram for displaying spatial infectious disease data and illustrating both incidence and absolute numbers of cases. A similar technique has been suggested before [12], but to our knowledge it was never used to study clustering of point patterns at a national level in infectious disease epidemiology. We compared the dot map cartogram to the frequently used choropleth incidence map and the dot map [1]. The main advantages of the dot map cartogram over the other two is that it is able to simultaneously reveal epidemic patterns adjusted for the population distribution, and to unmask patterns that are hidden by aggregation and categorisation of information. The visual distortion of the dot map cartogram is a barrier to pinpointing the location of a case: this is a benefit in the field of infectious diseases because case privacy should be ensured in presenting surveillance data. In addition, dot map cartograms illustrate areas without cases, which are harder to discern by the use of choropleth incidence maps.

We did not address map types other than dot maps and incidence maps for this comparison. Smooth incidence maps are an alternative to the choropleth incidence maps, in which the incidence is smoothed across the spatial units. This technique was applied to the data from a previous study for the Q fever outbreak in the Netherlands in 2009 [13]. In illustrating hotspots, the dot map cartogram is comparable to the smooth incidence map. The advantage of the smooth incidence map is that it permits identification of the exact location of hot spots, since the map is not deformed. However, smooth incidence maps do not reveal areas without cases and it is hard to discern the absolute number of cases and the true scope and size of the outbreak. Many more mapping techniques exist, such as map types based on other interpolation techniques (such as inverse distance weighting, kriging and trend surface fitting [2]), incidence based value-by-area cartograms [14,15], and many others. Assessment of all available methods was not within the scope of this paper.

We used the Gastner and Newman diffusion-based algorithm [4] to construct the cartogram. Alternative algorithms are available to construct contiguous cartograms [16-26]. We chose the Gastner and Newman algorithm because it performs well compared to the alternative algorithms in terms of quantitative measures such as the cartographic error, shape error and topology error [10], and qualitative measures such as subjective preferences and task performance [27]. Recent studies have proposed quantitative [10] and qualitative [27] measures for cartogram generation techniques. The development of standardised performance measures will allow objective ranking and selecting of cartogram algorithms.

With the demonstration of dot map cartograms, we provide public health professionals with an alternative spatial method for outbreak analysis. Firstly, we expect that dot map cartograms minimise misinterpretation of the data. Secondly, as demonstrated in this study, dot map cartograms protect the privacy of cases. Thirdly, dot map cartograms do not require expensive specialised GIS software, which facilitates use in settings with limited resources. The only requirements to construct dot map cartograms are free software, case reports with location data and access to population data as shapefiles. As there is a general trend for governments to make administrative population data publicly available, the latter is available for most countries. Finally, our method can easily be extended with information on covariates relevant for mapping: a relevant example in infectious disease surveillance or research is to colour the case dots for a specific attribute, such as age group, sex or vaccination status. Introducing other layers of detail or attributes further broadens the utility of this spatial method for use in infectious disease research and surveillance. However, as the technique is graphical rather than geographical, it does not replace a geographical information system explaining the impact of various geographical factors on the spread of a certain infectious disease.
